# Intratympanic gentamycine for Ménière’s disease: is there a selective vestibulotoxic effect?

**DOI:** 10.1007/s00405-020-05901-3

**Published:** 2020-03-30

**Authors:** András Molnár, Stefani Maihoub, Anita Gáborján, László Tamás, Ágnes Szirmai

**Affiliations:** Department of Otolaryngology and Head and Neck Surgery, Semmelweis University, Szigony u. 36., Budapest, 1083 Hungary

**Keywords:** Ménière’s disease, Intratympanic gentamycine, Hearing loss, Pure tone audiometry, Vertigo attacks

## Abstract

**Purpose:**

The aim of our study is to investigate the effectiveness and safety of the treatment, based on vertigo diaries and pure tone audiograms.

**Methods:**

The complete medical documentation of 105 definite patients suffering from Ménière’s disease was analyzed. In the studied group, nine patients were treated with intratympanic gentamycine. Long-term follow-up of the patients was carried out, using vertigo diaries, medical letters, anamnestic data, and pure tone audiograms. Audiometric results and vertigo complaints before and after treatment were contrasted using IBM SPSS V24 software.

**Results:**

Based on our analysis, vertigo attacks appeared significantly less often after gentamycine treatment [*p* < 0.001; Odds ratio 0.003 (95% CI 0.001–0.012)], which confirms the efficacy of the therapy. Pure tone stages before and after the application of gentamycine were contrasted using the Mann–Whitney *U* test. When comparing the audiometric results of long-term follow-ups by using the logistic regression, a statistically significant difference was observed between the treated and not treated groups [*p* = 0.001; Odds ratio 0.141 (95% CI 0.064–0.313)], and based on the survivorship curve hearing impairment was more common in the not treated group which also supports our results. Based on the non-parametric test, there was no significant difference (*p* = 0.84) between the pure-tone stages of the control group and of those treated with gentamycine.

**Conclusion:**

Our results indicate that intratympanic gentamycine is effective in controlling vertigo attacks, and there is no higher risk for hearing loss than in case of spontaneous progression of the disorder.

## Introduction

Ménière’s disease (MD) is an idiopathic disorder of the inner ear, characterized by vertigo attacks, sensorineural hearing loss, tinnitus, and vegetative symptoms [1]. Management of MD is based on conservative symptomatic treatment of vertigo attacks, prophylactic treatment and lifestyle counselling (e.g. salt-restricted diet). After using these therapeutic options, about 80% of the patients could be in remission. For medically intractable MD, local destructive medical treatment or destructive surgical management are recommended [2]. As a destructive medical treatment, aminoglycoside antibiotics are used in clinical practice, known as chemical vestibular ablation [3]. The procedure was first used by Schuknecht in 1957. Since he used streptomycin, vertigo spells were controlled, but SNHL occurred in 62% of the cases [4]. Gentamycine seems to be more vestibuloototoxic, because of the more damaging effect on vestibular hair cells versus cochlear ones [5], especially on type I hair cells [6]. Gentamycine is an aminoglycoside antibiotic with a well-known ototoxic effect [7], based on the increase in reactive nitrogen and oxygen species in the inner ear, resulting in dysfunction of hair cells [8]. Intratympanic therapies are widely used in the everyday clinical practice because local application allows higher concentrations in the perilymph with lower applied doses without systemic adverse effects [9, 10]. Intratympanic gentamycine (ITG) results in complete or incomplete ablation of the vestibular function, differences in the success of the therapy are based on the individuality of peripheral vestibular function and central compensation [11]. There are conflicting data in the literature about gentamycine induced hearing loss; some authors suggested no existence of hearing loss after ITG therapy, while in other studies the incidence of hearing loss varies from 3 to 45% of the cases [12, 13].

## Purpose

Because ITG treatment ideally should control vertigo attacks while preserving hearing, our study aims to investigate whether there is a selective vestibulotoxic effect or not.

## Materials and Methods

### Patients

The subjects of the present investigation were MD patients who were seen at the Department of Otolaryngology and Head and Neck Surgery of Semmelweis University from 2000 to 2019. In that period, 105 MD patients (31 males, 74 females, mean age ± SD, 57.38 years ± 11.07) were diagnosed in the tertiary referral neurotologic centre. All patients were diagnosed as having definite MD, according to the diagnostic criteria of the Bárány Society [14] as follows:

1. Two or more spontaneous episodes of vertigo, each lasting 20 min to 12 h.
2. Audiometrically documented low—to medium frequency sensorineural hearing loss in one ear, defining the affected ear on at least one occasion before, during or after one of the episodes of vertigo.
3. Fluctuating aural symptoms (hearing, tinnitus or fullness) in the affected ear.
4. Not better accounted for by another vestibular diagnosis.

ITG treatment was proposed to nine patients. The duration of the disorder before treatment was 33.67 ± 1.54 months (mean ± SD). Control patients were included from the 105 definite MD patients, who were not treated with ITG. Long-term follow-up of the patients was carried out, using vertigo diaries, medical letters, anamnestic data, and pure tone audiograms (PTA). Hearing loss was expressed as an extended Fletcher index (average of losses on 500, 1000, 2000 and 4000 Hz frequencies). All of the patients had a documented follow-up of at least 2 years, the mean ± SD follow-up time was defined as 67.76 ± 45.89 months. The research project had the consent of Semmelweis University Regional and Institutional Committee of Science and Research Ethics: 47/2018.

### Application of gentamycine

Intratympanic drug therapies are based on the passage of intratympanically administered drugs into the inner ear, which are administered through the tympanic membrane and by diffusion from the middle ear into the inner ear through the round window. The application of ITG was performed under a microscope, and with the patients in a supine position, with the affected ear upwards. After confirming the intact tympanic membrane and the status of the middle ear, local anaesthesia with 10% lidocaine pump spray was administered. After the anaesthetization, 8 mg gentamycine sulphate was instilled through the anteroinferior quadrant of the tympanic membrane. The procedure was carried out 2 to 4 times, on alternate days until acute unilateral vestibular hypofunction with typical clinical signs appeared. ITG treatment was used only in cases of unilateral MD. Before and 6–8 weeks after the treatment, pure tone audiometry was performed. ITG was indicated only in reasonable cases, only if the conservative management of symptoms for at least 6 months was unsuccessful, and if the quality of life deteriorated, based on the patients’ report. Only patients with at least 40 dB hearing loss on speech frequencies were treated with ITG.

### Statistical analysis

The statistical analysis was completed by using the IBM SPSS V24 software. As most of the studied parameters did not show normal distribution (based on Shapiro–Wilk test), a non-parametric test was used (Mann–Whitney *U* test). The significance level was specified as *p* < 0.05. To illustrate our results, survivorship curves, the so-called Kaplan–Meier curves were included as well. The result of the long-term follow-up was analyzed using the log-rank test as well.

## Results

**Fig. 1:**
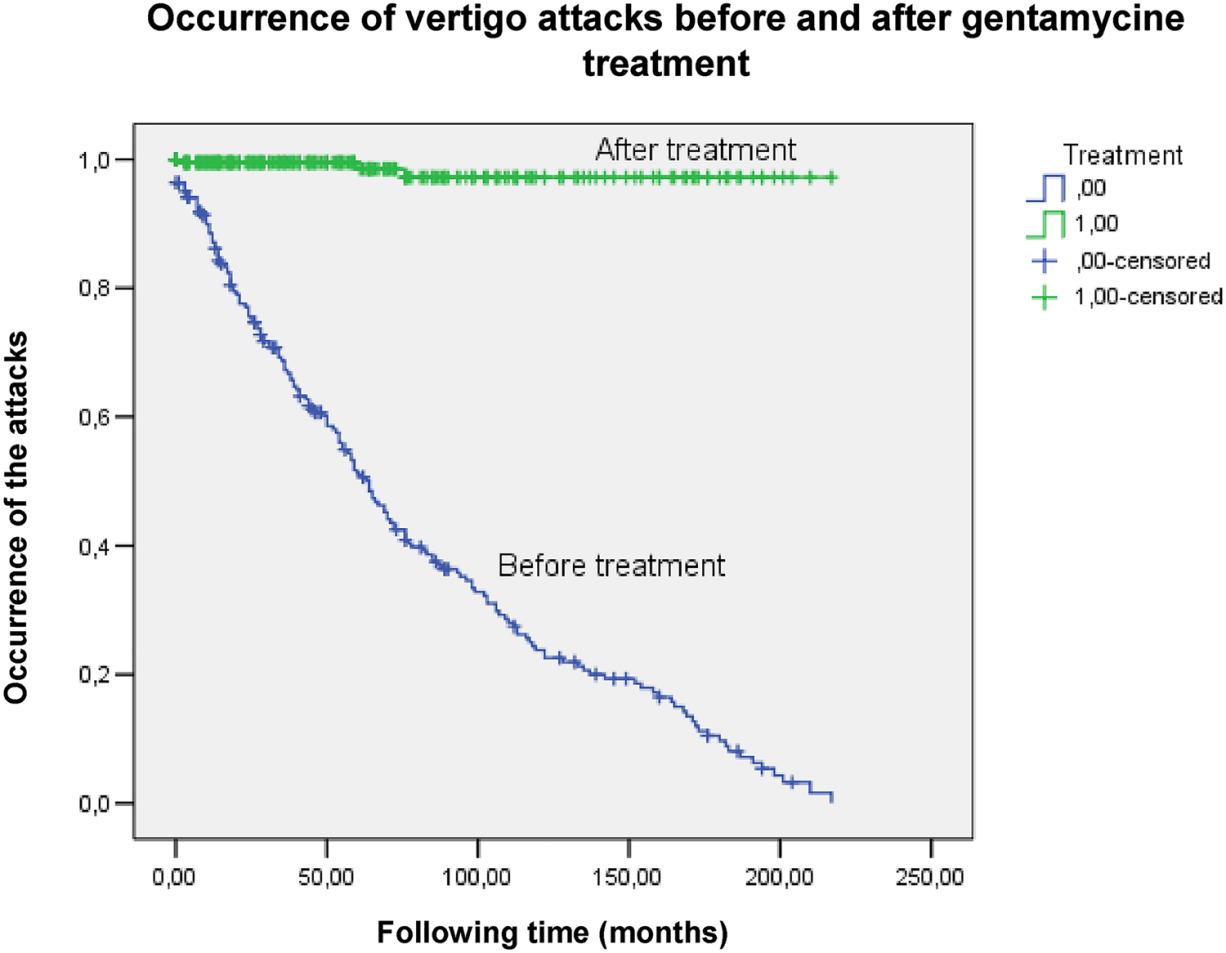
Occurrence of attacks before and after ITG treatment. In SPSS the number of events (i.e. vertigo attack) that happened at time (following time) was determined as follows: 0, if 3 or less attacks/month and 1, if more than 3 attacks/month. The vertical axis (Y) represents the probability of the defined event (i.e. vertigo attack): at 1.0 the probability of attack-free period is 100%, and when the curve drops down, the occurrence of vertigo attacks is more frequent

First, the occurrence of vertigo attacks before and after ITG treatment was analyzed, based on the long-term follow-up of the symptoms. The results are shown in the Kaplan–Meier curve (Fig. 1).

As shown in Fig. 1, vertigo attacks appeared significantly less often after ITG treatment, the result is also strengthened by the outcome of the long-rank test [*p* < 0.001; Odds ratio 0.003 (95% CI 0.001–0.012)], which implies a very strong statistically significant difference between the two groups. Based on the symptoms, vertigo control was complete in most of the cases, only two patients were complaining about attacks after treatment (one of them had two, the other one had one episode), but these were shorter and transient, not typical attacks for MD. From the data, it can be concluded that ITG treatment was effective in all of the cases, and no re-treatment was necessary.

To determine the possible cochleotoxic effects of ITG therapy, pre- and post-treatment pure tone audiograms were analyzed and contrasted. The results are shown in Table 1. A significant change in hearing was defined as follows: more than 10 dB change on the lower frequencies (125, 250, 500 and 1000 Hz) and more than 15 dB change on the higher ones (2000, 4000, 8000 Hz). As shown in the table, there were more positive changes detected, and there was an impairment seen that was not enough to be significant in most of the cases. Pure tone stages before and after the application of ITG were contrasted using the Mann–Whitney *U* test. As shown in the table, there was no statistically significant difference detected between pre-and post-treatment results in all cases, indicating no significant change in the hearing profile after ITG.

**Table 1:** Demographic and audiometric data of patients treated with ITG

	Age (years)	Gender (M: male, F: female)	Side (r: right, l: left)	Time between diagnosis and treatment (months)	Pure tone average (mean dB ± SD) before treatment	Pure tone average (mean dB ± SD) after treatment (3 months)	Difference (mean ± SD)	Frequencies of change [dB]	*p* value (Mann–Whitney *U* test)
1	65	F	L	93	65 ± 7.5	69.3 ± 9.2	4.29 ± 4.89	500 [+ 10], 2000 [+ 10], 4000 [+ 10]	0.53
2	66	F	L	60	81.7 ± 5.56	78.3 ± 5.56	3.33 ± 4.44	500 [− 10], 2000 [− 10]	0.52
3	64	F	R	9	45 ± 5.7	52.8 ± 6.9	7.86 ± 3.9	250 [− 10], 1000 [− 10], 2000 [− 10], 8000 [− 15]	0.13
4	59	F	L	14	50 ± 5	58.8 ± 6.9	8.75 ± 6.56	125 [+ 10], 250 [+ 10], 500 [+ 10], 4000 [+ 20], 8000 [+ 20]	0.08
5	76	F	L	21	85.7 ± 6.12	80 ± 2.85	5.7 ± 6.12	125 [+ 10], 250 [+ 10], 500 [+ 10], 1000 [+ 10], 2000 [− 10], 4000 [+ 10]	0.14
6	76	F	R	48	65.7 ± 7.75	68.6 ± 4.89	2.86 ± 6.12	125 [− 10], 250 [− 10], 1000 [+ 10], 2000 [− 10]	0.65
7	64	M	R	28	62.86 ± 8.98	61.43 ± 7.75	1.43 ± 4.9	500 [+ 10], 1000 [− 10], 2000 [+ 10]	0.85
8	50	F	R	37	58.57 ± 7.35	61.43 ± 4.9	2.86 ± 4.08	500 [− 10], 2000 [− 10]	0.57
9	38	F	L	10	52.86 ± 9.8	55.71 ± 16.7	2.86 ± 8.98	125 [+ 10], 250 [+ 10], 1000 [− 10], 2000 [− 20], 4000 [− 10]	0.95

+ indicates improvement, while – impairment

**Fig. 2:**
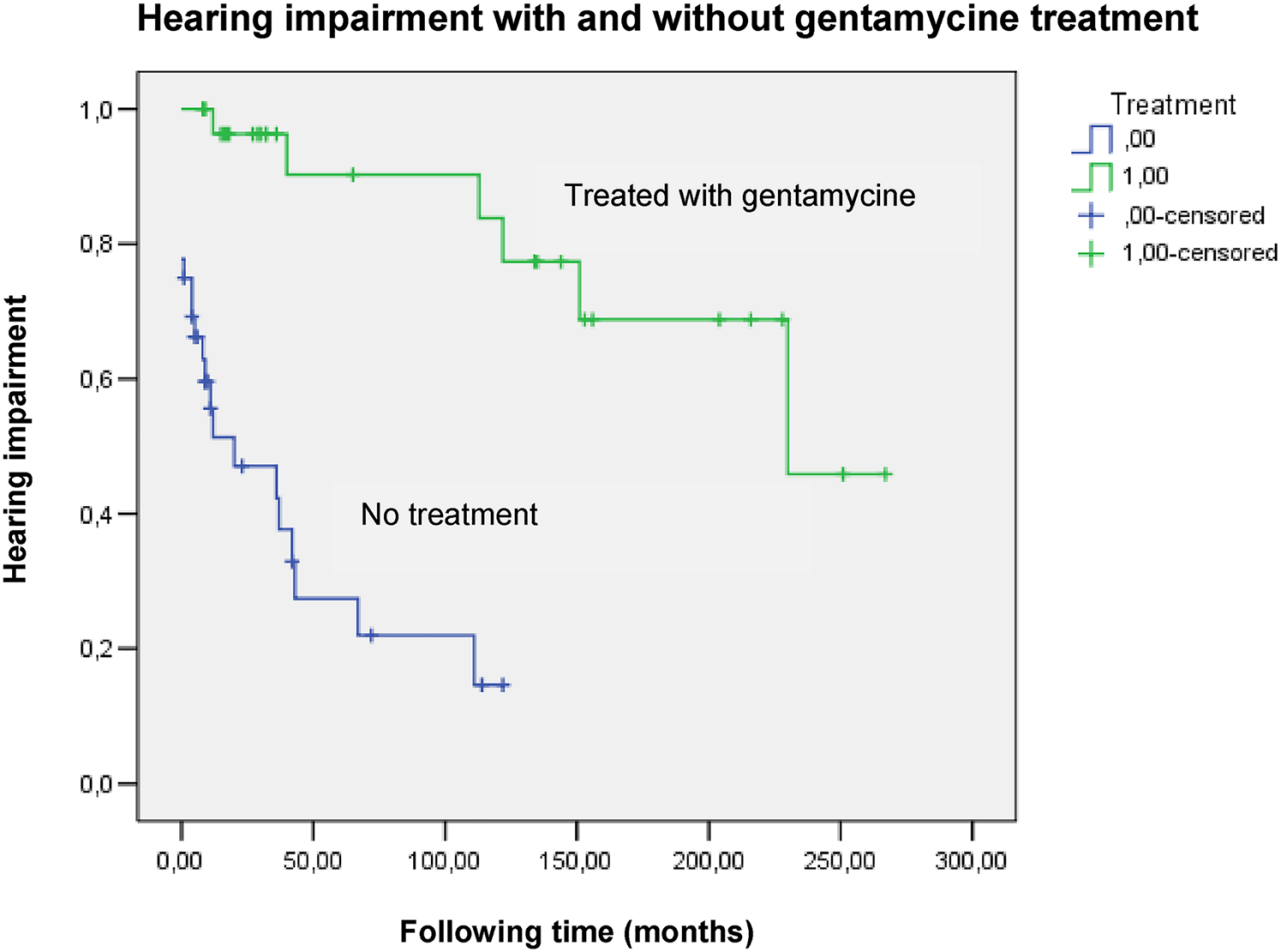
Kaplan Meier curve to determine the long-term effects of ITG. To analyze the connection between hearing loss and ITG therapy, patients were divided into two groups: treated with gentamycine or not. No treatment group is consisting of PTA results of patients before they were treated with ITG and of those from the control group (MD patients who were not treated). In SPSS, the number of events (i.e. hearing impairment) that happened at time (following time) was determined as follows: 0, if no significant change contrasted to the previous PTA examination was observed; 1 if significant change on at least 2 frequencies was detected

The long-term effects of ITG on hearing was analyzed too (Fig. 2).

As shown in Fig. 2, hearing impairment occurred to be more common in the no treatment group, indicating that using ITG is not resulting in a higher risk of hearing loss than the spontaneous progression of the disorder. When compared by using logistic regression, a statistically significant difference was observed between the treated and not treated groups [*p* = 0.001; Odds ratio 0.141 (95% CI 0.064–0.313)], which also supports our results.

**Fig. 3:**
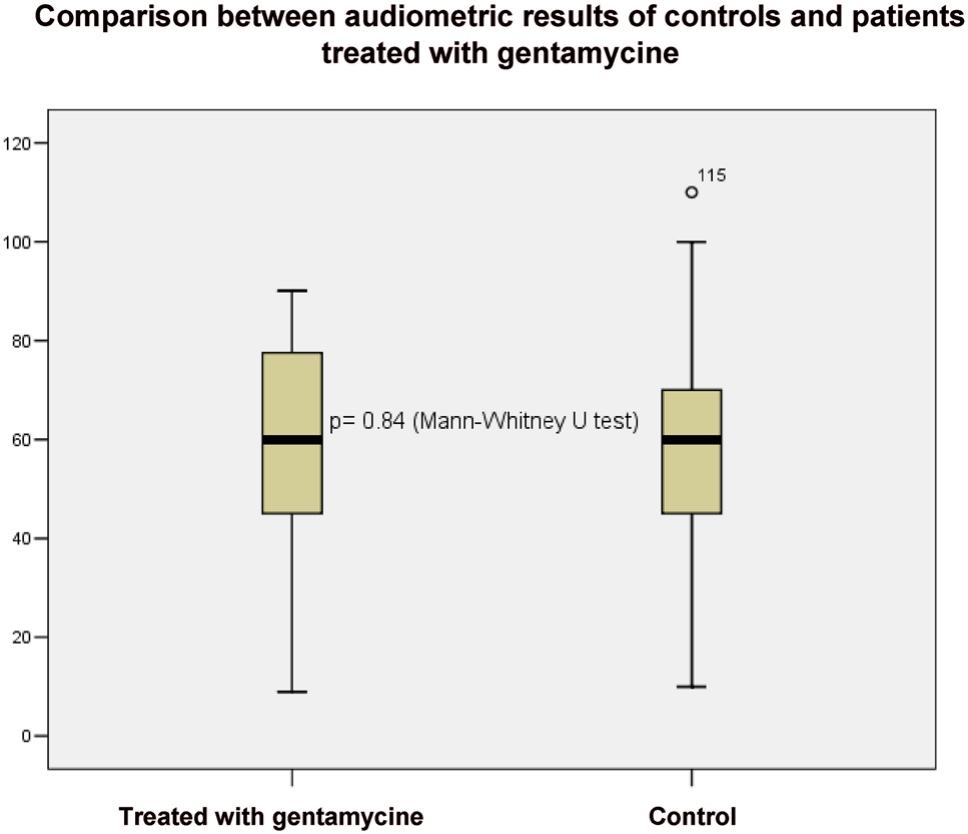
Boxplot about the comparison of the PTA results of patients treated with gentamycine and controls

Audiometric results of control patients who were not treated with ITG and of those treated were contrasted as well. Results are shown in the boxplot (Fig. 3).

As shown in Fig. 3, there is no obvious difference between the two boxes, and the result of statistical analysis (*p* = 0.84) also does not suggest a difference between treated (mean ± SD, 58.76 ± 15.14 dB) and control (57.37 ± 14.51 dB) groups. To analyze results in a more detailed way, low and high frequency stages were contrasted separately. Based on statistical analysis, no significant difference was determined, neither in case of lower frequencies (*p* = 0.49) nor in case of higher ones (*p* = 0.1).

## Discussion

Conservative treatment for MD is efficient in most of the cases, intratympanic gentamycine is necessary only for intractable MD. In the investigated population, ITG was performed on nine patients (8.57%). Previously the effectiveness of ITG was analyzed by some authors. The results of Rolf J. Postema et al. demonstrated that vertigo score did not change in the control group, but it has been decreased in the group of the ITG treatment [15]. In the study of Nicolas Perez et al. it has been concluded that vertigo was controlled in 83% of the patients, and recurrence of complaints was noted in 20.5% [3]. In our study complete control was achieved in all cases, only three vertigo attacks appeared in two patients, but these were not typical MD attacks, were transient, and no re-treatment was necessary. Beck and Schmidt stated that no complete ablation of vestibular function is necessary to reduce vertigo symptoms [16]. The importance of that fact is that lower, and not daily applicated doses of gentamycine is enough, just like the dosage characteristic we use in our clinical expertise. That is also important to protect cochlear functions.

In clinical practice, the risk of sensorineural hearing loss is often a reason why doctors providing the treatment hesitate to use ITG in reasonable cases. ITG is an effective therapy for intractable MD cases; however, it is also important to try to eliminate vertigo attacks while cochlear functions should be preserved. That is the reason why our study is focused on the possible hearing loss after an ITG treatment.

Of course, the occurrence and frequency of side effects depend on the applied doses of ITG as well. This effect has been investigated by a retrospective study of Casani et al. There were two groups compared; one was treated with higher (2 mL of 27.6 mg/mL concentration, six times) and one with lower (only one injection) doses of ITG. In the group treated with lower doses, not only hearing outcomes were better but also the effectiveness of the therapy on the control of vertigo [17]. In a previous study, a complete vertigo control of 43 patients was reported after ITG therapy, and with the preservation of the hearing status, with almost no change detected in the cochlear function [12]. Earlier studies indicate that higher doses of ITG, with daily injections with or without titration result in significantly worse outcomes of hearing loss after treatment, 33.4% [18], 45% [19] and 57% [20] of post-treatment sensorineural hearing loss were reported. In later studies, when lower doses were used in weekly intervals, the frequency of hearing loss was lower, for example, 17% [21] and 12% [22]. However, the complete reduction of vertigo symptoms was a little bit less too (70% and 81%). Based on our data, it can be concluded that our protocol with doses applied on a non-daily basis, caused the complete reduction of complaints while preserving hearing status after treatment. Previously in a review study, it has been stated that 2.1 of ITG treatment is necessary to reduce symptoms on average [23]. In our population, this ratio was defined as 3.3 ± 0.56 (range 2–4 times).

## Conclusion

This study was conducted to investigate the audiovestibular impact of ITG treatment. Although many studies can be found about the results in the literature, further investigation is necessary because of the high variety of the frequency of control of vertigo attacks and hearing loss, and applied protocols and doses according to them. Based on our data, it can be concluded that with our protocol, ITG is a potent and safe treatment option of intractable MD.
